# Adapting to Regional Enforcement: Fishing Down the Governance Index

**DOI:** 10.1371/journal.pone.0012832

**Published:** 2010-09-17

**Authors:** Henrik Österblom, U. Rashid Sumaila, Örjan Bodin, Jonas Hentati Sundberg, Anthony J. Press

**Affiliations:** 1 Fisheries Centre, The University of British Columbia, Vancouver, Canada; 2 Baltic Nest Institute, Stockholm Resilience Centre, Stockholm University, Stockholm, Sweden; 3 Stockholm Resilience Centre, Stockholm University, Stockholm, Sweden; 4 Department of Systems Ecology, Stockholm University, Stockholm, Sweden; 5 Antarctic Climate and Ecosystems Cooperative Research Centre, Hobart, Tasmania, Australia; University of California Davis, United States of America

## Abstract

**Background:**

Illegal, Unreported and Unregulated (IUU) fishing is a problem for marine resource managers, leading to depletion of fish stocks and negative impacts on marine ecosystems. These problems are particularly evident in regions with weak governance. Countries responsible for sustainable natural resource management in the Southern Ocean have actively worked to reduce IUU fishing in the region over a period of 15 years, leading to a sequence of three distinct peaks of IUU fishing.

**Methodology/Principal Findings:**

We reviewed existing public records relating to IUU fishing in the Southern Ocean between 1995–2009 and related this information to the governance capacity of flag states responsible for IUU vessels. IUU operators used a number of methods to adapt to enforcement actions, resulting in reduced risks of detection, apprehension and sanctioning. They changed fishing locations, vessel names and flag states, and ports for offloading IUU catches. There was a significant decrease in the proportion of IUU vessels flagged to CCAMLR countries, and a significant decrease in the average governance index of flag states. Despite a decreasing trend of IUU fishing, further actions are hampered by the regional scope of CCAMLR and the governance capacity of responsible states.

**Conclusions/Significance:**

This is the first study of long-term change in the *modus operandi* of IUU fishing operators, illustrating that IUU operators can adapt to enforcement actions and that such dynamics may lead to new problems elsewhere, where countries have a limited capacity. This outsourcing of problems may have similarities to natural resource extraction in other sectors and in other regions. IUU fishing is the result of a number of factors, and effectively addressing this major challenge to sustainable marine resource extraction will likely require a stronger focus on governance. Highly mobile resource extractors with substantial funds are able to adapt to changing regulations by exploiting countries and regions with limited capacity.

## Introduction

Resilient organizations are characterized by a high adaptive capacity, often resulting from well developed social networks which maintain and build trust and social capital [1], [2]. Such institutions have been described for the management of natural resources [3], but organizations involved in criminal activities have also been described as resilient to law enforcement due to a high adaptive capacity [4]. Sustainable management of complex and dynamic natural resources benefit from resilient governance structures that can adapt to changing conditions [5], but such management can be severely complicated by different forms of environmental crime [6]. Illegal, Unregulated and Unreported (IUU) fishing [7], [8], [9] is a challenge for sustainable management of marine ecosystem and regions with weak governments may be particularly vulnerable to IUU fishing [8]. Thus far, relatively little is known about factors that contribute to the resilience of IUU fishing and how IUU operators adapt to changing conditions.

Here, we investigate the adaptive capacity of IUU fishing by documenting 15 years of changing *modus operandi* by vessel operators involved in such fishing in the Southern Ocean. The aim of this study is to describe how IUU fishing changed over time in the CCAMLR (the Commission for the Conservation of Antarctic Marine Living Resources) Convention area, and how these changes influence the capacity of CCAMLR to effectively address this non-compliance, using proxies for flag state governance capacity. Flag states hold the primary responsibility for vessels [10], but their incentives, authority or ability to address non-compliance [11] may depend on a range of factors.

Previous research has shown that efforts to address IUU fishing in one region can move the problem elsewhere, or create other novel problems [12], [13]. These characteristics, where any attempt to solve a problem will generate new ones, is analogous to the definition of wicked problems, which have no ultimate solution [14]. Another definition of a wicked problems is that they are usually symptoms of other problems [14]. Overcapacity and subsidies [9], [15], [16], depleted fish stocks [17], perceived lack of legitimacy of remote fishing zones [15], poverty and lack of alternative livelihoods [9], have all been described as contributing to IUU fishing.

Substantial IUU catches for toothfish *Dissostichus* spp. have been recorded in the Southern Ocean. Although IUU catches of other species and in other regions are more substantial [8], the long history (>15 years) of IUU fishing for *Dissostichus* spp. combined with the dedicated work by members (Contracting Parties: CPs) of the regional management body (CCAMLR) provides a valuable platform for studying the dynamics of IUU fishing and strategies employed by vessel owners and operators to maintain their resilience to actions taken by flag states, port states and countries with nationals involved in such fishing. Toothfish stocks are concentrated around Sub-Antarctic islands in the Southern Ocean and a number of CCAMLR CPs are involved in regulated fisheries for toothfish, which has provided a strong incentive for them to reduce IUU fishing [13], [18], [19], [20], [21].

When IUU fishing emerged as a major issue for CCAMLR in the mid 1990s, its IUU catches was several times higher than those of licensed fisheries and threatened the sustainability of toothfish stocks and seabird populations (as a direct result of bycatches in the longline fishery for toothfish) [22]. Because of its unreported nature, the level of IUU fishing compromised the accuracy of toothfish stock assessments and impacted the profitability of the toothfish industry [23]. Estimated economic losses from IUU fishing for toothfish are in the order of hundreds of million U.S. dollars [24], [25]. Vessels engaged in IUU fishing have been recorded as relocating from South America and Europe as a result of stock depletion of toothfish and other predatory fish stocks elsewhere [19]. Information on IUU fishing during the early 2000s indicated an increased involvement of Asian interests and the emergence of organized and coordinated trade of toothfish in Asia [25]. Several suspected IUU vessels included refitted former tuna long-lining vessels. New, and more valuable, purpose built vessels were increasingly being used as the IUU fishing industry developed. Efforts to address IUU fishing has been complicated by the ability of IUU vessel operators to change vessel name, flag state and fishing location. This general trend has been described elsewhere [13], [20].

In this study, we show that actions taken by CCAMLR and CPs to address IUU fishing have resulted in adaptations by actors engaged in IUU fishing. These adaptations have increased the resilience of IUU fishing, thereby complicating the work of CCAMLR by creating a wicked problem.

## Results

### A sequence of adaptations by IUU operators

A total of 147 suspected offshore IUU activities involving 72 officially identified vessels were recorded between 1995–2009. Out of these records, 95% were related to IUU fishing and 5% related to support of known IUU vessels.

**Figure 1:**
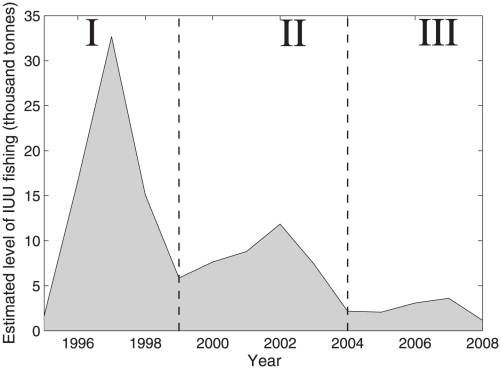
Three periods of IUU fishing in the Southern Ocean. Estimated levels of IUU fishing in the CCAMLR area between 1995–2008 [50] during three distinct phases.

**Figure 2:**
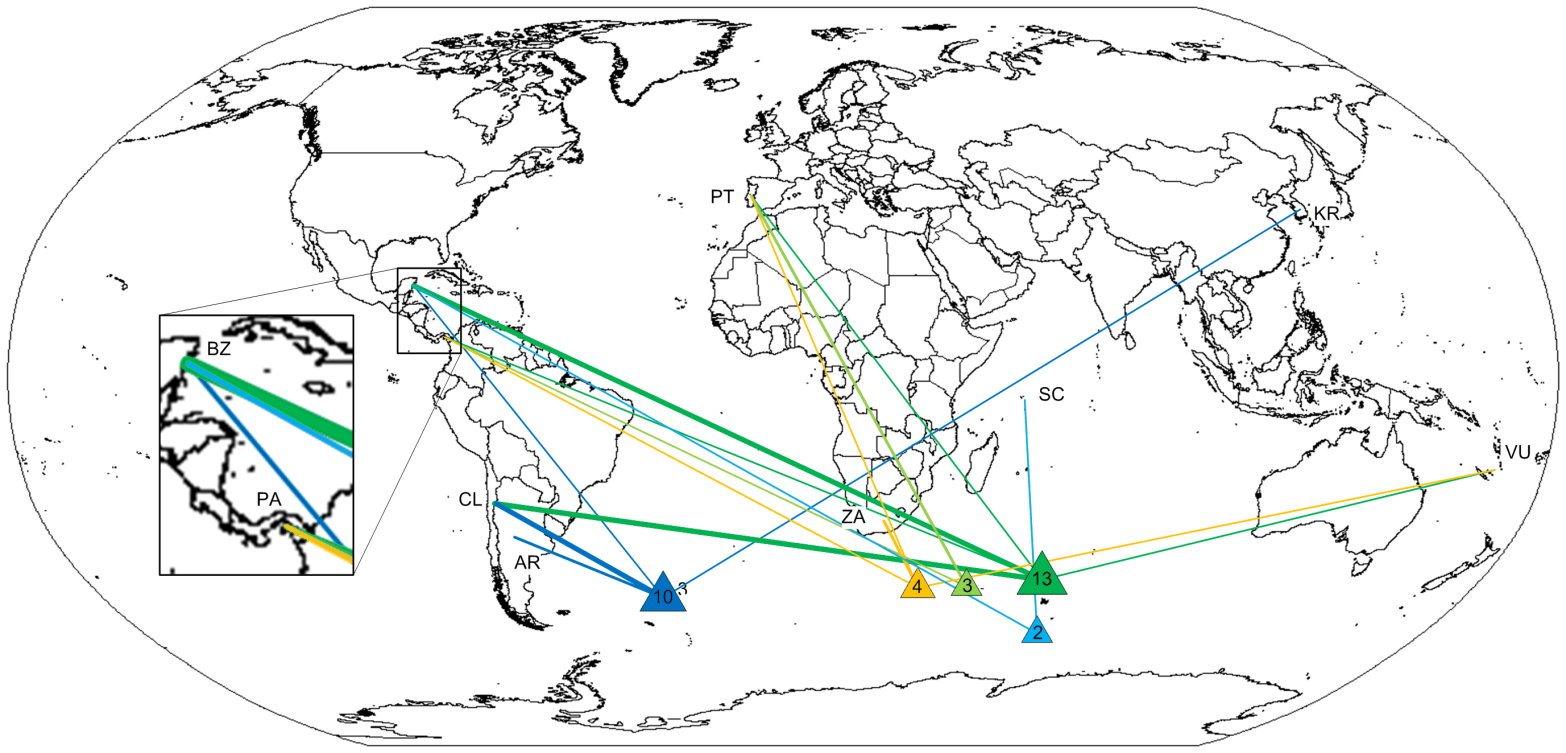
Changing fishing locations and flag states 1995–1999. Suspected IUU vessels of known location and flag states during the first period (see Figure 1). Argentina (AR), Belize (BZ), Chile (CL), Republic of Korea (KR), Panama (PA), Portugal (PL), Seychelles (SC), South Africa (ZA). Thin line: one record, medium line: two records, thick line: three or more records.

During the first period (1995–1999), estimated IUU fishing reached a historical peak and also declined substantially (Fig. 1). 39 records involved 31 vessels and 23% of the vessels were observed more than once. 97% of all records contained information on the location of the IUU activity. No name changes were recorded, although one flag changed during the period. Information on flag states (97% of all records) showed that CCAMLR CPs Argentina (24%) and Chile (16%), as well as Non-Contracting Parties (NCPs) Belize (21%), Panama (13%) and Portugal (11%), dominated the list of flag states (Fig. 2). Relatively little information was available on the nationality of officers (23%) and crew (15%), but the data suggests substantial involvement of Spanish (56%) and Chilean officers (22%) during this period. Crews were from nine countries, mainly in Southern Africa, South America and Spain. Port records indicated a preference for offloading IUU catches of toothfish in Port Louis, Mauritius (69%, n = 64), and Walvis Bay, Namibia (23%), as well as other Southern African harbors. The average landed amount was 208 tonnes (n = 29), including both fishing vessels and cargo ships.

**Figure 3 pone-0012832-g003:**
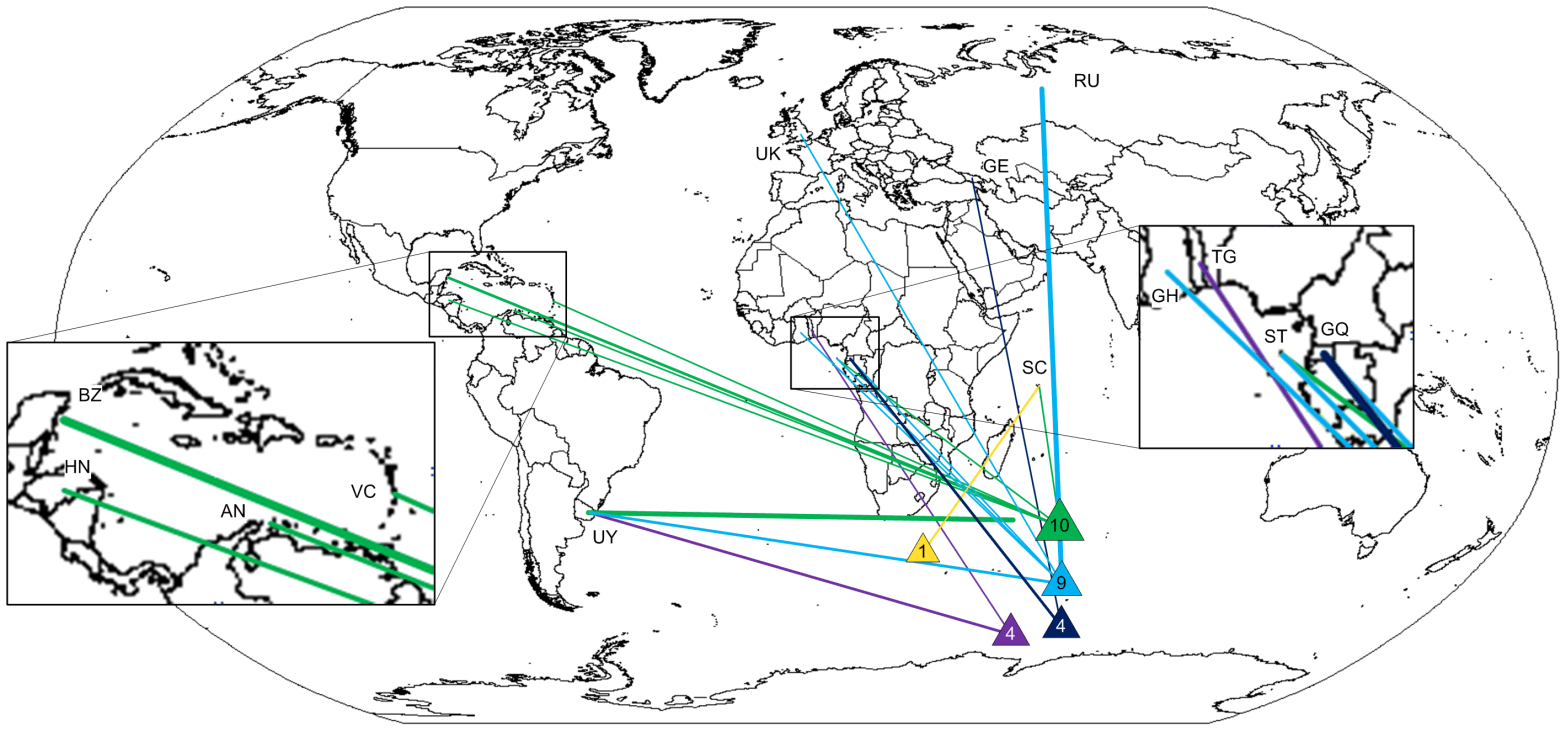
Changing fishing locations and flag states 2000–2004. Suspected IUU vessels of known location and flag states during the second period (see Figure 1). Netherlands Antilles (AN), Belize (BZ), Georgia (GE), Ghana (GH), Equatorial Guinea (GQ), Honduras (HN), Russia (RU), Seychelles (SC), Sao Tome and Principle (ST), Togo (TG), United Kingdom (UK), Uruguay (UY), Saint Vincent and Grenadines (VC).

During the second period 41 incidents involved 30 vessels, with 27% of all vessels being observed more than once. Three of these were also active in the previous period. Five name and flag changes were recorded. Records with information on flag states (95%) showed that CCAMLR CPs Uruguay (23%) and Russia (13%) had emerged as new major flag states, together with 4 other CPs and 10 NCPs. No vessels were flagged to either Chile or Argentina (Fig. 3). 83% of the records contained information on suspected IUU location. Information on the nationality of officers and crew was available for 37% and 22% of the records respectively. Spanish nationals were officers in all known records, although Russian and Uruguayan officers were also identified. Crewmembers were mainly from Chile and Indonesia. Mauritius was still an important harbor for suspected IUU landings (36%, n = 28 records), as was Walvis Bay (21%), and a number of Southern African ports, and for the first time, East Asian (Indonesia, Malaysia, Singapore) ports. An average of 209 tonnes toothfish per vessel was offloaded (n = 11).

**Figure 4:**
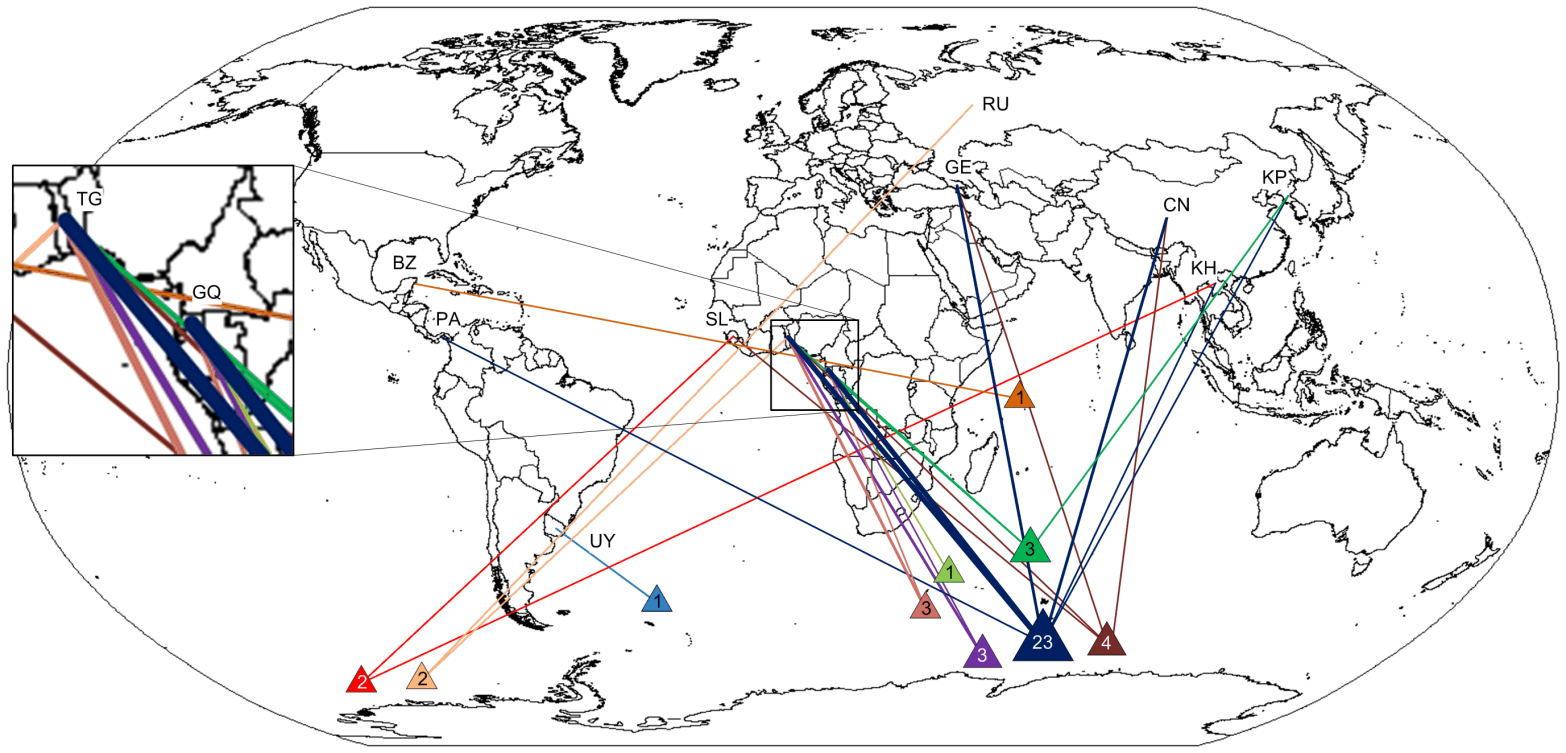
Changing fishing locations and flag states 2005–2009. Suspected IUU vessels of known location and flag states during the third period (see Figure 1). Belize (BZ), China (CN), Georgia (GE), Equatorial Guinea (GQ), Cambodia (KH), Democratic People's Republic of Korea (KP), Panama (PA), Russia (RU), Sierra Leone (SL), Togo (TG).

During the third period (2005–2009), estimated IUU catches were lower than previously (Fig. 1), but dedicated offshore patrol programs generated substantial information (67 records of 26 vessels, with 62% of vessels observed more than once). Half of all vessels observed (13 vessels) had been active in the previous period, but only one of these had also been active during the first period. The location of IUU fishing activity was known in 94% of the records. Seventeen name changes and 9 flag changes were recorded during the period. The available information on flag states (91%) illustrated China as flag state (8%), although before it became a Contracting Party to CCAMLR. NCPs Togo (38%) and Equatorial Guinea (23%) emerged as important flag states. Two of the vessels recorded engaged in IUU activities with flags from these countries have previously been documented as IUU vessels flagged to Contracting Parties. An additional six vessels on the CCAMLR IUU list have also reflagged from Contracting Parties to Togo or Equatorial Guinea at some point, although the publicly available records did not contain any information on suspected IUU activities of these vessels while flagged to contracting parties, prior to reflagging to Togo or Equatorial Guinea. A number of vessels were also flagged to North Korea (8%), (Fig. 4). Spanish and Chilean nationals were involved as officers and crew in the few records available (6%). There was only a single recorded suspected landing of IUU toothfish during the period. An additional 28 records of port visits by vessels known to have been involved in IUU fishing for toothfish were recorded, but no information regarding landings of toothfish was available for these vessels.

### Temporal trends in governability

**Figure 5:**
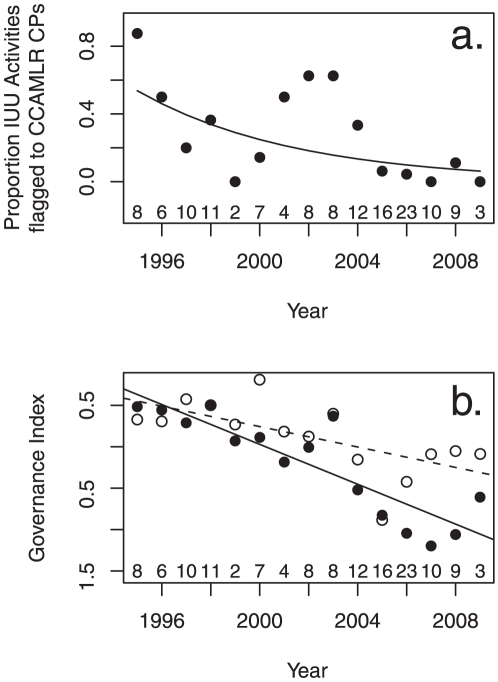
The capacity of CCAMLR to address IUU fishing. Changes over time in a) The proportion of suspected offshore IUU activities with vessels flagged to CCAMLR contracting parties (CP), y = e∧(306.8677−0.1541*yr) and b) Flag state governance capacity. Solid symbols = Average, combined governance index for Government Effectiveness, Regulatory Quality, Rule of Law and Control of Corruption, of flag states for all suspected IUU activities, y = 240.82−0.12 * yr. Open symbols = Governance indexes for Stability/No violence, of flag states for suspected offshore IUU activities, y = 125.51−0.062 * yr. The number of suspected IUU activities recorded each year is indicated in the graph.

The number of IUU fishing vessels flagged by a particular flag state generally increased over a short time period and subsequently decreased, presumably as vessels involved in IUU fishing were deregistered by the flag state. New flag states emerged over time and generally showed a similar pattern of flagging IUU fishing vessels. There was a significant negative trend in the proportion of IUU fishing activities associated with vessels flagged to CCAMLR CPs over time (log-transformed data, R^2^ 0.32, p<0.02, n = 15, Fig. 5a) as vessels increasingly used flags of NCPs.

The mean values of four governance indices of flag states (Government Effectiveness, Regulatory Quality, Rule of Law and Control of Corruption, see [8]) declined significantly over time (R^2^ 0.75, p<0.001, n = 15, Fig. 5b), indicating that vessels are not only using NCP flags to a larger extent, but are also increasingly choosing countries with weak governments as flag states, with, presumably, lower ability to address non-compliance. There was also a significant decrease in an additional index for governance (Stability/No violence) over time (R^2^ 0.38, p<0.01, n = 15), however, the slope was significantly smaller than for the other governance indices (p<0.03, Fig. 3b.) which could indicate a preference in ship owners of using relatively stable countries as flag states.

## Discussion

### Effects of actions to address non-compliance

Estimated levels of IUU fishing have decreased substantially from 1995 to 2009, illustrating progress made to address IUU fishing by CCAMLR and CPs [13], [15], [19], [23], [26]. The three pulses of IUU fishing appear to correspond to a number of adaptations in *modus operandi* of IUU fishing operators, although we also observed a continuous adaptation as measured in governance capacity of flag states (Fig. 5). IUU vessels have increasingly been pushed out of national waters of the UK, France and Australia due to increased monitoring, control and surveillance at sea, and from South African waters due to stock depletion [13], [19], [27], [28]. This has led to a change in fishing locations (an eastward and southward expansion of the IUU fishery and increased fishing in the high seas [13], [19], [20], and increased targeting of Antarctic toothfish, *D. mawsoni*, rather than Patagonian toothfish, *D. eleginoides*. Such roving bandit behavior [29] has been described for marine resources elsewhere [17], but primarily as a response to stock depletion, rather than enforcement efforts.

Some suspected vessels were merely observed and reported, but not apprehended – the suspected IUU activities thus had no direct consequences for those with interests in the vessels. Several of the apprehended vessels however, were bonded for release. These vessels were subsequently either released as the owners paid the bond, or remained the property of apprehending governments, which sold, scrapped or refitted the vessel for other purposes. Released vessels may have relocated to other areas and other fisheries.

CCAMLR CPs have for the most part, actively de-flagged their vessels when they have been identified as engaged in IUU activities. NCPs have experienced diplomatic pressure from CCAMLR CPs [15], resulting in actions against vessels flagged to these countries. However, diplomatic actions towards the more recently emerging IUU vessel flag states appear less effective, as their response to requests by CCAMLR members is limited [26]. Over the period of this study, the pattern of preferred IUU vessel flag states has changed, from primarily vessels initially being flagged to CPs, then to NCPs, and, in the recent period, increasingly to flag states with weak governance. The capacity of CCAMLR to further reduce IUU fishing is hampered by the fact that none of the recorded IUU fishing vessels currently active are flagged to CPs, and their current flag states have limited capacity to take action against their vessels, or are not effectively in control of their vessel register. The recent concentration of IUU fishing vessels flagging to a small number of flag states may indicate that a decreasing number of countries are willing to flag IUU vessels, or that the operators and beneficial owners of these vessels are deliberately targeting weak governance.

Several states have developed national legislation aimed at dissuading their nationals from engaging in IUU fishing [30]. Some countries have been successful in reducing the involvement of their nationals [23] and other have more recently made such progress [26], [28]. For instance, a number of legal procedures are underway in Spain [31]. Recent European legislation and ongoing developments in Chilean legislation is directly aimed at reducing the involvement of related nationals in IUU fishing [26]. However, effectively addressing nationals involved in IUU fishing requires a dialogue between relevant countries and flag states, and this dialogue can be severely hampered when these flag states have a low governance capacity.

Taking action against IUU vessel owners is often not straightforward, as ownership is commonly disguised by complex company structures, often in part registered in tax havens [32]. However, significant diplomatic pressure, as well as social pressure from environmental NGOs and industry has been directed at vessels owners [23], [25], [28], [33], [34], [35], [36] with some success.

Ports of landings have also changed over time. Mauritius was unequivocally identified as the main port for IUU-caught fish during the first period, and was also important during the second period [37]. Together with Walvis Bay, Namibia, these ports were eventually closed down to recorded IUU landings [37], [38]. Recent public information on port landings is generally lacking, although the CCAMLR Commission refers to, for example, landings in Singapore [28], the largest container port in the world [39]. Australia has recently further referred to Indonesia, Malaysia, Mozambique and Kenya as important ports for IUU vessels [28].

### Adaptive capacity of IUU fishing

Presumably changes in *modus operandi* of IUU fishing aims at reducing the risks and costs of detection, apprehension, and sanctioning. The Australian hot pursuit of the Russian flagged *Lena* in 2002 (which managed to escape as she was refueled by another vessel [40]) and the apprehension of the same *Lena* and *Volga* that year, suggested that IUU fishing had become increasingly organized [24]. The *Volga* case before the International Tribunal for the Law of the Sea (ITLOS) revealed that she was fishing in a fleet of at least seven boats. These vessels allegedly received logistic support from an oil tanker, as well as detailed information on the whereabouts of the Australian fisheries patrol vessel [41]. The master of the *Lena* also suggested that *Lena* and *Volga* (the oldest vessels in the fleet) were sacrificed in order to prevent the other more valuable vessels from being apprehended [41].

In addition to frequent name and flag changes, other means to reduce the probability of detection [7] included obscuring the name of the vessel [42]. Means to reduce the probability of apprehension, once detected, include receiving legal advice during hot pursuit [15]. If detected, standard procedure appears to have included the dumping of catch, log books, computers and other potential evidence [42]. Sustained legal support during the trials of apprehended IUU operators reduces the probability of sanctioning, and are also indicative of coordinated activities [43]. A trial and conviction related to IUU fishing for toothfish exposed the existence of bribery of port officials [44], a technique used to further reduce the risk of detection.

A large number of vessels identified during the first period were not observed in subsequent periods. Agnew [19] suggested that a hard core of IUU operators had replaced many of the opportunistic operators active during the first period. Currently active IUU operators, fishing in the high seas and flagged to NCPs, are beyond the reach of CCAMLR inspections (that is, they are engaged in unregulated fishing). The recent concentration of vessels flagged to Togo and Equatorial Guinea indicate that this is currently a viable *modus operandi* option. These countries are among the lowest scoring for several governance indices [45]. However, they score relatively high on indices related to stability and violence, indicating that IUU operators tend to choose stable flag states with limited governance capacity.

The ports of landing IUU catches appear to be increasingly far from the fishing grounds. This would result in higher costs of IUU fishing (larger steaming distances), however many of the vessels currently on the CCAMLR IUU vessel lists have been observed in harbor without catch [27], and it has been suggested that they increasingly conduct transshipments at sea to cargo vessels or to legitimately licensed fishing vessels [27], [40]. IUU fishing vessel operators have also started to use gillnets for catching toothfish [28], [46]. This is likely to reduce costs and also generate valuable bycatch [26], [46]. Estimated catches with this gear type is uncertain.

All indications suggest that the IUU fishing fleet inside the CCAMLR convention area has decreased and that no new vessels are being observed [26]. Whether these vessels have ceased their IUU activities or are developing new ones elsewhere [46], is currently uncertain.

IUU operators appear adaptive, likely due to a combination of increased offshore coordination and consolidation, hidden corporate beneficiaries and substantial monetary assets - factors that enable them to effectively reduce risks and increase profits. These factors contribute to their resilience and thus the wickedness of the problem of combating IUU fishing in the Southern Ocean. The substantial profits for vessel owners and officers have produced strong incentives to maintain and develop their capacity to remain resilient in the face of change.

### Potential biases

The information on suspected IUU fishing provided here suffer from a number of potential biases (see Materials and Methods). The conclusions may therefore be skewed towards changes in *modus operandi* of IUU fishing operators more likely to be detected, whereas IUU fishing operators less known and less detected will be under-represented. Nonetheless, the substantial information collected here, over an extended time period, we argue, is sufficiently reliable to provide valuable insights into some key adaptations made by IUU operators in response to CCAMLR and individual States enforcement measures, although acknowledging that care should be taken when interpreting the results. Other adaptations than those described here are likely to have occurred. For instance, the name and flag changes reported here is incomplete, as we only record changes between known records of IUU activities. The IUU vessel list maintained by CCAMLR [47] keep updated records of black-listed vessel since 2003, including all known name and flag changes. This list indicates the adaptive capacity of several well-known IUU vessels that have operated in the region continuously since 2003.

### Conclusions

A high adaptive capacity has enabled IUU fishing vessel operators to continue to engage in IUU fishing in the CCAMLR area despite enforcement measures and international pressure on flag states. There appears to be no clear ultimate solutions to IUU fishing, as attempts to solve it generate new problems elsewhere (thereby making IUU fishing correspond to the definition of a wicked problem). In the CCAMLR area, IUU operators adapted by changing fishing ground, ports of landings and flag states. This adaptive capacity has resulted in a co-evolution between IUU operators and managing authorities at national and international levels. The magnitude of IUU fishing in the EEZ of a country has been correlated to governance capacity of that state [8]; and compliance mechanisms in a Regional Fisheries Management Organization (RFMO) can lead to the displacement of IUU vessels [12]. Here, we show that changing flag states appear to be a deliberate strategy aimed at adapting to management measures.

National and regional approaches to IUU fishing taken in isolation risk the perverse result of ‘outsourcing’ the problems to regions and countries with less ability to address them. This “race to the bottom”, or “fishing down the governance index” with a relocation of IUU fishing operations to regions with weak governance, can only be addressed by realizing that IUU fishing is a symptom of other problems. Addressing non-compliance and novel adaptations in IUU fishing must build on enforcement and management measures at appropriate scales, including, for example, implementation of the recent port state agreement [12], [48], information sharing, capacity building (for example using the IMCS network, www.imcsnet.org), and increased international coordination [8]. However, addressing overcapacity, depleted fish stocks, RFMO legitimacy and alternative livelihoods for crew on IUU vessels would also provide complementary approaches to direct action on IUU fishing.

## Materials and Methods

We reviewed existing public records relating to the dynamics of IUU fishing in the Southern Ocean between 1995–2009. Records of alleged IUU activities (fishing, other offshore activities, landings) were derived from publicly available documents from CCAMLR and governments, national and international court cases, report from fisheries inspections, reports and web pages from NGOs and the fishing industry, journal articles, press releases and books (Tables S1 and S2).

### Three phases of IUU fishing

CCAMLR continuously estimate the levels of IUU fishing for toothfish each fishing season (December 1–November 30), and these estimated suggest that IUU fishing have exhibited three distinct phases (Fig. 1). We used the fishing seasons (or CCAMLR-years) these three phases to define our three periods for investigating changes in modus operandi of IUU fishing, using November 30 as break point in 1999 and 2004 respectively. We make the case that the three phases are the results of adaptations taken by IUU operators in response to (among other things) diplomatic pressure, a Catch Documentation Scheme and increased offshore enforcement, respectively [13], [15], [19], [49]. The aim of separating the data into these periods is to investigate general patterns of adaptations by IUU fishing operators.

The number of recorded IUU activities reported for these periods is not individually comparable, due to different levels of intensity in the publication of, for example, NGO reports or offshore patrols (detecting IUU fishing location, as well as vessel name and flag changes). Rather, the information obtained is intended to illustrate major trends in vessel flags, nationality of crew, landing ports, and other general changes in *modus operandi*. Main fishing locations derived from these records are illustrated, although these estimates are based on less detailed information than CCAMLR estimated IUU catches [50].

This study uses data and information from publicly available resources and these resources containing information on suspected IUU activities suffer from a number of potential biases. Vessels with operators more skilled at avoiding detection and apprehension will be less likely to be included in these sources. Conversely, well-known IUU fishing vessels will have the eyes of industry, environmental NGOs and government agencies on them, resulting in more records for these vessels. Fishing locations, flag states and nationality of crew for IUU vessels may also influence the probability of being recorded in the material used. Fishing locations less monitored will result in fewer records. Information on IUU fishing from countries used as flag states or having their nationals involved in IUU fishing may be influenced by e.g. the activity of environmental NGOs in that country, where countries with active NGO groups or fishing industry will be more likely to provide information on IUU fishing. The proportion of all IUU activities being recorded may also vary in space and over time due to changing focus of groups and organization providing information on IUU fishing. Vessels may change name, flag, color marking and other distinguishing features, resulting in a risk that any vessel recorded twice under different names will be recorded as two vessels. In this study, substantial efforts have been made to use all available information in public records to follow all known name and flag changes.

Not all alleged IUU fishing activities in these records have resulted in apprehension and/or sanctioning. Some allegations of IUU activities has also been challenged by the flag states of suspected vessels, e.g., [27]. However, they represent the best available publicly recorded synthesis of suspected IUU activities in the CCAMLR area. All vessels included in the description of trends for catch location, flag states, and officer and crew nationality, have either been sanctioned or identified as suspected IUU vessels in official documents from CCAMLR, international (ITLOS, International Tribunal for the Law of the Seas) or national court proceedings, or in public documents from governments. Records from NGO reports or newspaper articles referring to sightings of vessels without such official records are not included. Information on suspected IUU ports are, however, drawing on additional sources (Table S2) and include vessels that have not been officially alleged to be engaged in IUU activities. The recorded landings of toothfish are from vessels not licensed to fish by CCAMLR and/or landing toothfish without proper catch documentation [19]. Only details on locations and amounts are included from the reports of suspected IUU landings and any information on vessel name, flag state and crew has been omitted.

### Governance capacity

The capacity of CCAMLR to effectively address IUU fishing between 1995–2009 is investigated using two different proxies: a) the proportion of all records with vessels flagged to CCAMLR contracting parties (CP); and b) changes in governance indexes [45] of flag states of identified vessels. Here, we use regular year for observations of IUU activities and corresponding governance proxies. The proportion of recorded IUU activities by vessels flagged to CCAMLR Contracting Party (CP) states were divided by the total number of recorded activities for CPs and NCPs to investigate to what extent IUU activities was associated with CCAMLR. We assumed that non-compliance of vessels flagged to CCAMLR CPs would be easier to address than vessels flagged to NCPs. We also assigned two corresponding governance indexes for each IUU activity in any given year. One index used the methodology of Agnew [8] and pooled the governance indexes for Government Effectiveness, Regulatory Quality, Rule of Law and Control of Corruption. We also used an additional index for Violence/Stability of flag states. In the absence of an index for a particular year, we used a mean value of adjacent years, or the closest value for records prior to, or after existing indices. We averaged all obtained governance indexes each year, which was correspondingly weighted to the number of observations in the analysis.

## Supporting Information

Table S1 Sources for information on suspected offshore IUU activities: vessel location, name, flag state and crew.
(0.15 MB DOCX)

Table S2 Sources for information on port visits.
(0.14 MB DOCX)
