# Supplementary material for: Adapting to Regional Enforcement: Fishing Down the Governance Index
Source: PLoS One. 2010 Sep 17;5(9):e12832. doi: 10.1371/journal.pone.0012832 (PMC2941461; doi:10.1371/journal.pone.0012832)
Supplement: Table S1 — Sources for information on suspected offshore IUU activities: vessel location, name, flag state and crew. (0.15 MB DOCX) [file pone.0012832.s001.docx]

**Table S1.** Sources for information on suspected offshore IUU activities: vessel location, name, flag state and crew

| **Year** | **Reference** |
| --- | --- |
| 1995 | [1,2] |
| 1996 | [2,3] |
| 1997 | [3,4,5,6,7,8,9,10,11,12,13,14,15,16,17,18] |
| 1998 | [4,5,6,7,8,9,10,11,12,13,15,16] |
| 1999 | [7,11,12,15,17,18,19,20,21,22,23] |
| 2000 | [5,6,7,11,13,14,15,16,17,24,25,26,27,28,29,30] |
| 2001 | [6,7,11,14,15,17,28] |
| 2002 | [5,11,12,13,15,16,19,20,23,25,26,28,31,32,33,34,35] |
| 2003 | [7,11,13,20,26,34,36,37,38,39,40,41,42,43] |
| 2004 | [5,6,11,13,16,23,26,42,44,45,46,47,48,49,50] |
| 2005 | [5,7,11,13,26,39,40,45,51,52,53,54,55,56,57,58] |
| 2006 | [26,37,38,40,41,52,53,58,59] |
| 2007 | [26,38,45,54,60,61,62] |
| 2008 | [26,52,63,64,65,66,67,68,69] |
| 2009 | [26] |

1. CCAMLR (1995) Report of the Fourteenth Meeting of the Commission. Hobart: CCAMLR.

2. CCAMLR (1996) Report of the Fifteenth Meeting of the Commission. Hobart: CCAMLR.

3. Dodds K (2000) Geopolitics, Patagonian Toothfish and Living Resource Regulation in the Southern Ocean. Third World Quarterly 21: 229-246.

4. CCAMLR (1998) Report of the Seventeenth Meeting of the Commission. Hobart: CCAMLR.

5. Griggs L, Lugten G (2007) Veil Over The Nets: Unravelling Corporate Liability For IUU Fishing Offences. Marine Policy 31: 159-168.

6. Baird R (2004) Illegal, unreported and unregulated fishing: an analysis of the legal, economic and historical factors relevant to its development and persistence. Melbourne Journal of International Law 5: 299-334.

7. Fallon LD, Kriwoken LK (2004) International Influence of an Australian Nongovernmental Organization in the Protection of Patagonian Toothfish. Ocean Development & International Law 35: 221-226.

8. ISOFISH (1998) The involvement of Mauritius in the trade in Patagonian toothfish from illegal and unregulated longline fishing in the Southern Ocean and what might be done about it. ISOFISH Occasional Report No. 1. Hobart: ISOFISH.

9. ISOFISH (1998) The Vikings: The involvement of Norwegian fishermen in illegal and unregulated longline fishing for Patagonian toothfish in the Southern Ocean. ISOFISH Occasional Report No. 3. Hobart: ISOFISH.

10. ISOFISH (1999) The Chilean fishing industry: Its involvement in and connections to the illegal and unregulated exploitation of Patagonian toothfish in the Southern Ocean. ISOFISH Occassional Report No. 2. Hobart: ISOFISH.

11. Peyron M (2010) The on-going tooth-fish saga in the Southern Ocean. Accessed: <http://homepage.mac.com/jmdelacre/tribune/page3/page3.html>. Available 2010 June 1.

12. Greenpeace (2010) Sibley (Sunk). Database of fishing vessels and companies engaged in Illegal, Unregulated and Unreported (IUU) fishing: Available: <http://blacklist.greenpeace.org/0/vessel/show/122-sibley-sunk>. Accessed 2010 June 1.

13. ASOC (2005) ASOC Red List - April 2005. ASOC.

14. CCAMLR (2001) Report of the Twentieth Meeting of the Commission. Hobart: CCAMLR.

15. DAFF (2005) Australian National Plan of Action to Prevent, Deter and eliminate Illegal, Unreported and Unregulated Fishing. Canberra: Australian Government, Department of Agriculture, Fisheries and Forestry.

16. Roche J-M (2010) L'histoire du patrouilleur Albatros (1995-...). Netmarine: Available: <http://www.netmarine.net/bat/patrouil/albatros/histoire3.htm>. Accessed 2010 June 1.

17. Anonymous (2010) Norwegian Black List. Bergen: Directorate of Fisheries. Available: <http://www.fiskeridir.no/english/fisheries/norwegian-black-list>. Accessed 2010 June 1.

18. ITLOS (2000) The “Camouco” case (Application for prompt release): Public sitting held on Thursday, 27 January 2000, at 15.00 hours at the International Tribunal for the Law of the Sea, Hamburg. Hamburg: ITLOS.

19. CCAMLR (2002) Report of the Twenty-First Meeting of the Commission. Hobart: CCAMLR.

20. CCAMLR (2003) Report of the Twenty-Second Meeting of the Commission. Hobart: CCAMLR.

21. CCAMLR (1999) Report of the Eighteenth Meeting of the Commission. Hobart: CCAMLR.

22. Anonymous (2000) 'White Gold' - the Pirate Menace Earth Report: TVE.

23. Anonymous (2004) Fisheries News. Mercosur: Mercopress. Accessed: <http://en.mercopress.com/2004/06/30/fisheries-news>. Available 2010 June 1.

24. Molenaar EJ (2004) Multilateral Hot Pursuit and Illegal Fishing in the Southern Ocean: The Pursuits of the Viarsa 1 and the South Tomi. International Journal of Marine and Coastal Law 19: 19-42.

25. Knecht GB (2006) Hooked: Pirates, Poaching and the Perfect Fish: Rodale.

26. CCAMLR (2010) Combined IUU vessel list adopted from 2003 to 2009. CCAMLR. Available: <http://www.ccamlr.org/pu/e/sc/fish-monit/iuu-vess.htm>. Accessed 2010 June 1.

27. ITLOS (2000) The “Monte Confurco” case (Application for prompt release): Public sitting held on Thursday, 7 December 2000, at 15.15 p.m., at the International Tribunal for the Law of the Sea, Hamburg,. Hamburg: International Tribunal for the Law of the Sea.

28. NET (2004) Black Market for White Gold: the Illegal Trade in Chilean Sea Bass. National Environmental Trust.

29. Greenpeace (2010) Greenpeace Gallery of Toothfish Vessels. Amsterdam: Available: <http://archive.greenpeace.org/oceans/southernoceans/expedition2000/gallery/pirates.html>. Accessed 2010 June 1.

30. CCAMLR (2000) Report of the Nineteenth Meeting of the Commission. Hobart: CCAMLR.

31. ITLOS (2002) Minutes of Public Sitting held on 12, 13 and 23 December 22. The "Volga" Case (Russian Federation v. Australia), Prompt Release. Hamburg: International Tribunal for the Law of The Sea.

32. Greenpeace (2010) Gold Dragon. Database of fishing vessels and companies engaged in Illegal, Unregulated and Unreported (IUU) fishing. Available: <http://blacklist.greenpeace.org/0/vessel/show/119-gold-dragon>. Accessed 2010 June 1.

33. COLTO (2003) Rogues Gallery: The new face of IUU fishing for toothfish. COLTO. Available: <http://www.colto.org/PDFs/RoguesGallery.pdf>. Accessed 2010 June 1.

34. Greenpeace (2010) Amorinn. Database of fishing vessels and companies engaged in Illegal, Unregulated and Unreported (IUU) fishing. Amsterdam: Available: <http://blacklist.greenpeace.org/0/vessel/show/114-amorinn>. Accessed 2010 June 1.

35. Masters C (2002) The Toothfish Pirates. Australia: Australian Broadcasting Corporation.

36. Gianni M, Simpson W (2005) The Changing Nature of High Seas Fishing - How flags of convenience provide cover for illegal, unreported and unregulated fishing. Australian Department of Agriculture, Fisheries and Forestry, International Transport Workers’ Federation, and WWF International. .

37. Greenpeace (2010) Perseverance. Database of fishing vessels and companies engaged in Illegal, Unregulated and Unreported (IUU) fishing. Amsterdam: Available: <http://blacklist.greenpeace.org/0/vessel/show/121-perseverance>. Accessed 2010 June 1.

38. CCAMLR (2007) Report of the Twenty-Sixth Meeting of the Commission. Hobart: CCAMLR.

39. CCAMLR (2005) Report of the Twenty-Fourth Meeting of the Commission. Hobart: CCAMLR.

40. CCAMLR (2006) Report of the Twenty-Fifth Meeting of the Commission. Hobart: CCAMLR.

41. Greenpeace (2010) Aldabra. Database of fishing vessels and companies engaged in Illegal, Unregulated and Unreported (IUU) fishing. Amsterdam: Available: <http://blacklist.greenpeace.org/0/vessel/show/113-aldabra>. Accessed 2010 June 1.

42. Anonymous (2004) COLTO claims "Maya V", "Viarsa 1" link. Mercopress Available: <http://enmercopresscom/2004/01/30/colto-claims-maya-v-viarsa-1-link> Accessed 2010 June 1.

43. Greenpeace (2010) Heavy Sea. Database of fishing vessels and companies engaged in Illegal, Unregulated and Unreported (IUU) fishing: Available: <http://blacklist.greenpeace.org/0/vessel/show/118-duero>. Accessed 2010 June 1.

44. Greenpeace (2010) Greenpeace exige al Gobierno copias de los Expedientes relacionados con los buques de Vidal Armadores, S.A. Madrid: Available: <http://www.greenpeace.org/espana/news/greenpeace-exige-al-gobierno-c>. Accessed 2010 June 1.

45. MARM (2009) Actuaciones del MARM en relación con determinados buques implicados en pesca ilegal en aguas reguladas por la CCAMLR. Madrid: Ministerio de Medio Ambiente y Medio Rural y Marino. Available: <http://www.mapa.es/gabinete/nota.asp?codi=20820_AT050309>. Accessed 2010 June 1.

46. Anonymous (2006) Judgement: Case no. A19/2006. Durban: High Court of South Africa: Durban and Coast Local Division.

47. Anonymous (2004) Last three "Maya V" crewmembers fined and deported. Mercopress Available: <http://enmercopresscom/2004/11/18/last-three-maya-v-crewmembers-fined-and-deported> Accessed 2010 June 1.

48. Anonymous (2005) Fisheries hot news. Mercosur: Mercopress. Available: <http://en.mercopress.com/2005/10/04/fisheries-hot-news>. Accessed 2010 June 1.

49. CCAMLR (2004) Report of the Twenty-Third Meeting of the Commission. Hobart: CCAMLR.

50. MARM (2006) La Secretaría General de Pesca Marítima tiene abierto un expediente al buque Galaecia por transbordo de suministros durante una accíon piloto experimental. Madrid: Ministerio de Medio Ambiente y Medio Rural y Marino. Accessed: <http://www.mapa.es/gabinete/nota.asp?codi=4023_AT070406>. Available 2010 June 1.

51. Jacobsen G (2006) Toothfish poachers unlikely to pay fines. The Sydney Morning Herald. Sydney: Fairfax Digital. Available: <http://www.smh.com.au/news/national/toothfish-poachers-unlikely-to-pay-fines/2006/09/27/1159036580637.html>. Accessed 2010 June 1.

52. CCAMLR (2008) Report of the Twenty-Seventh Meeting of the Commission. Hobart: CCAMLR.

53. Greenpeace (2010) Draco-1. Database of fishing vessels and companies engaged in Illegal, Unregulated and Unreported (IUU) fishing. Amsterdam: Available: <http://blacklist.greenpeace.org/0/vessel/show/116-liberty>. Accessed 2010 June 1.

54. Carnie T (2007) Vessel fined for carrying gill nets. Mercury. Durban: Mercury & Independent Online (Pty) Ltd. Available: <http://www.iuufishing.org/index.php?option=com_content&task=view&id=18&Itemid=31>. Accessed 2010 June 1.

55. Greenberg P (2005) The Catch. The New York Times. New York: Available: <http://www.nytimes.com/2005/10/23/magazine/23bass.html?pagewanted=all>. Accessed 2010 June 1.

56. Greenpeace (2010) South Ocean. Database of fishing vessels and companies engaged in Illegal, Unregulated and Unreported (IUU) fishing: Available: <http://blacklist.greenpeace.org/0/vessel/show/110-south-ocean>. Accessed 2010 June 1.

57. Greenpeace (2010) North Ocean. Database of fishing vessels and companies engaged in Illegal, Unregulated and Unreported (IUU) fishing. Amsterdam: Available: <http://blacklist.greenpeace.org/0/vessel/show/109-north-ocean>. Accessed 2010 June 1.

58. Greenpeace (2010) West Ocean. Database of fishing vessels and companies engaged in Illegal, Unregulated and Unreported (IUU) fishing. Amsterdam: Available: <http://blacklist.greenpeace.org/0/vessel/show/112-west-ocean>. Accessed 2010 June 1.

59. Greenpeace (2010) Constant. Database of fishing vessels and companies engaged in Illegal, Unregulated and Unreported (IUU) fishing. Amsterdam: Available: <http://blacklist.greenpeace.org/0/vessel/show/129-constant>. Accesed 2010 June 1.

60. MRAG, Capfish (2008) Study and Analysis of the Status of IUU fishing in the SADC Region and an Estimate of the Economic, Social and Biological Impacts - Main Report. Gaborone: Stop Illegal Fishing.

61. Greenpeace (2010) Good Hope. Database of fishing vessels and companies engaged in Illegal, Unregulated and Unreported (IUU) fishing. Amsterdam: Available: <http://blacklist.greenpeace.org/0/vessel/show/127-toto>. Accessed 2010 June 1.

62. Greenpeace (2010) Triton-I. Database of fishing vessels and companies engaged in Illegal, Unregulated and Unreported (IUU) fishing. Amsterdam: Available: <http://blacklist.greenpeace.org/0/vessel/show/128-triton-I>. Accessed 2010 June 1.

63. Greenpeace (2009) Greenpeace pide a la Audiencia Nacional que investigue a una empresa española por pesca ilegal. Madrid: Available: <http://www.greenpeace.org/espana/news/090305>. Accessed 2010 June 1.

64. Losada S (2009) The new EU regulation on IUU fishing: A NGO perspective 5th Update and Stakeholder Consultation on Illegal, Unreported and Unregulated (IUU) Fishing Chatham House, London.

65. CCAMLR (2009) Report of the Twenty-Eight Meeting of the Commission. Hobart: CCAMLR.

66. Greenpeace (2010) EXPOSED! Pirates bankrolled by Spanish government. Amsterdam: Available: <http://www.greenpeace.org/international/news/exposed-pirates-bankrolled-by>. Accessed 2010 June 1

67. Anonymous (2008) Omunkete Fishing (PTY) Ltd v Minister of Fisheries (No 2), High Court of New Zeeland. Available: <http://www.maritimelaw.org.nz/0608.html>. Accessed 2010 June 1.

68. Greenpeace (2010) Trosky. Database of fishing vessels and companies engaged in Illegal, Unregulated and Unreported (IUU) fishing. Amsterdam: Available: <http://blacklist.greenpeace.org/0/vessel/show/156-paloma-v>. Accessed 2010 June 1.

69. Anonymous (2008) El palangrero que ardió en el Índico zozobró finalmente a 486 millas de Madagascar. La Voz de Galicia Available: <http://wwwlavozdegaliciacom/galicia/2008/07/11/0003_6976837htm> Accessed 2010 June 1.
