# Supplementary material for: Adapting to Regional Enforcement: Fishing Down the Governance Index
Source: PLoS One. 2010 Sep 17;5(9):e12832. doi: 10.1371/journal.pone.0012832 (PMC2941461; doi:10.1371/journal.pone.0012832)
Supplement: Table S2 — Sources for information on port visits. (0.14 MB DOCX) [file pone.0012832.s002.docx]

**Table S2**. Sources for information on port visits

| **Time period** | **References** |
| --- | --- |
| 1995-1999 (1^st^ period) | [1,2,3,4,5,6] |
| 2000-2004 (2^nd^ period) | [5,7,8,9,10,11,12,13,14] |
| 2005-2009 (3^rd^ period) | [15,16,17] |

1. Album G (1997) Fisket etter Patagonian Toothfish og norske interesser (Fishing for Patagonian toothfish and Norwegian interests). Oslo: Norges Naturvernforbund (Friends of the Earth, Norway). Available: <http://naturvernforbundet.no/cgi-bin/naturvern/imaker?id=116130>. Accessed 2010 June 1.

2. ISOFISH (1998) The involvement of Mauritius in the trade in Patagonian toothfish from illegal and unregulated longline fishing in the Southern Ocean and what might be done about it. ISOFISH Occasional Report No. 1. Hobart: ISOFISH.

3. ISOFISH (1998) The Vikings: The involvement of Norwegian fishermen in illegal and unregulated longline fishing for Patagonian toothfish in the Southern Ocean. ISOFISH Occasional Report No. 3. Hobart: ISOFISH.

4. ISOFISH (1999) The Chilean fishing industry: Its involvement in and connections to the illegal and unregulated exploitation of Patagonian toothfish in the Southern Ocean. ISOFISH Occassional Report No. 2. Hobart: ISOFISH.

5. Greenpeace (2010) Greenpeace Gallery of Toothfish Vessels. Amsterdam: Available: <http://archive.greenpeace.org/oceans/southernoceans/expedition2000/gallery/pirates.html>. Accessed 2010 June 1.

6. ITLOS (2000) The “Camouco” case (Application for prompt release): Public sitting held on Thursday, 27 January 2000, at 15.00 hours at the International Tribunal for the Law of the Sea, Hamburg. Hamburg: ITLOS.

7. CCAMLR (2000) Report of the Nineteenth Meeting of the Commission. Hobart: CCAMLR.

8. CCAMLR (2002) Report of the Twenty-First Meeting of the Commission. Hobart: CCAMLR.

9. CCAMLR (2003) Report of the Twenty-Second Meeting of the Commission. Hobart: CCAMLR.

10. CCAMLR (2004) Report of the Twenty-Third Meeting of the Commission. Hobart: CCAMLR.

11. Peyron M (2010) The on-going tooth-fish saga in the Southern Ocean. Accessed: <http://homepage.mac.com/jmdelacre/tribune/page3/page3.html>. Available 2010 June 1.

12. Anonymous (2005) Fisheries hot news. Mercosur: Mercopress. Available: <http://en.mercopress.com/2005/10/04/fisheries-hot-news>. Accessed 2010 June 1.

13. Gianni M, Simpson W (2005) The Changing Nature of High Seas Fishing - How flags of convenience provide cover for illegal, unreported and unregulated fishing. Australian Department of Agriculture, Fisheries and Forestry, International Transport Workers’ Federation, and WWF International. .

14. ASOC (2005) ASOC Red List - April 2005. ASOC.

15. CCAMLR (2006) Report of the Twenty-Fifth Meeting of the Commission. Hobart: CCAMLR.

16. CCAMLR (2007) Report of the Twenty-Sixth Meeting of the Commission. Hobart: CCAMLR.

17. PEW (2010) Port State Performance. The Pew Environment Group. Available: <http://portstateperformance.org/>. Accessed 2010 June 1.
